# Dual Antioxidant Supplementation With N-Acetylcysteine and Taurine (Nefrosave^®^) for Cardio-Renal Protection in Chronic Kidney Disease Patients With Diabetes and/or Hypertension: A Real-World, Retrospective, Comparative Study

**DOI:** 10.14740/cr2234

**Published:** 2026-08-31

**Authors:** Rama Kumari, Gayatri Veeramani Jayaraman, Soumya Kanti Dutta, Arindam Pande, M. Vijayakumar, V.S.R. Bhupal, Pankaj Jariwala, Priya Palimkar, Sandip Rungta, Devesh Rajani, Gautam Banerjee, Shobhnath Kumar, Durga Das Kothari, Jain Lalchand Tolchand, Priti Panchal, N. Nagashankar

**Affiliations:** a Nizam’s Institute of Medical Sciences, Hyderabad, Telangana 500082, India; b Mass General Brigham Hospital System, Boston, MA 02115, USA; c Kolkata Heart Foundation, Kolkata, West Bengal 700025, India; d Manipal Hospital, Kolkata, West Bengal 700099, India; e Vijaya Hospital, Chennai, Tamil Nadu 600026, India; f Capital Hospital, Vijayawada, Andhra Pradesh 521137, India; g Yashoda Hospital, Hyderabad, Telangana 500082, India; h Sahyadri Super Speciality Hospital, Pune, Maharashtra 411014, India; i Manipal Hospital, Kolkata, West Bengal 700029, India; j Medical Care Centre and Hospital, Lucknow, Uttar Pradesh 226005, India; k Kolkata Municipal Corporation, Kolkata, West Bengal 700013, India; 1 Maha Medical, Lucknow, Uttar Pradesh 226001, India; m Kothari Clinic, Kolkata, West Bengal 700037, India; n Jain Maternity and Nursing Home, Mumbai, Maharashtra 400069, India; o Lifewave Hospital, Mumbai, Maharashtra 400097, India; p Fourrts India Laboratories, Chennai, Tamil Nadu 600096, India

**Keywords:** Chronic kidney disease, N-acetylcysteine, Taurine, Microalbuminuria, Diabetes mellitus, Hypertension, Oxidative stress

## Abstract

**Background:**

Chronic kidney disease (CKD) is a growing global health burden. Type 2 diabetes mellitus (T2DM) and hypertension are predominant etiological factors. Oxidative stress plays a pivotal role, making antioxidant therapies promising. This study evaluated the effectiveness and tolerability of the antioxidant-combination N-acetylcysteine (NAC; 150 mg) and taurine 500 mg (Nefrosave^®^) in patients with CKD having T2DM and/or hypertension.

**Methods:**

This real-world, retrospective, multicentric study compared electronic medical records (EMRs) of adults with stages 1–3 CKD treated for 12 ± 2 weeks with NAC + taurine plus standard of care (n = 300; test) or standard of care alone (n = 150; control). Endpoints included changes in urinary albumin-to-creatinine ratio (uACR) and serum creatinine (primary), and changes in estimated glomerular filtration rate (eGFR) and adverse events (AEs, secondary).

**Results:**

Mean uACR reduced significantly in the test group (−8.61%; P < 0.001), but increased significantly in the control (+11.26%; P < 0.001). A similar trend was observed for changes in median serum creatinine (−0.05 mg/dL, P < 0.001 vs. +0.15 mg/dL, P < 0.001). eGFR improved in the NAC + taurine group, concomitant with a significant between-group difference of +6 mL/min/1.73 m^2^ (P < 0.001). Nephroprotective effects were consistent and favorable across CKD stages. A comparatively higher proportion of patients in the test group achieved ≥ 30% improvement in uACR and serum creatinine, although absolute proportions were low. AEs were infrequent and mild; no serious events occurred.

**Conclusion:**

NAC + taurine plus standard of care was more effective than standard of care alone in reducing uACR and improving eGFR. Tolerability was good with minimal AEs, supporting the NAC + taurine combination as a potent nephroprotective therapy in CKD patients having T2DM and/or hypertension.

## Introduction

Chronic kidney disease (CKD) is a major public health problem globally, affecting an estimated 850 million people and contributing substantially to cardiovascular morbidity, mortality, and healthcare costs [1, 2]. CKD is defined as persistent structural and/or functional kidney abnormality for at least 3 months which frequently progresses to end-stage kidney disease, if not detected and treated early [3, 4]. The burden is disproportionately higher in low and middle-income countries [5], with India and China together accounting for nearly one-third of the global CKD burden [1, 6]. The Indian CKD study identified diabetes and hypertension as the most common etiology for CKD in the country [7].

Diabetes and hypertension act synergistically on the renal microvasculature, promoting glomerular hyperfiltration, endothelial dysfunction, and progressive nephron loss [8]. Microalbuminuria, defined as a urinary albumin-to-creatinine ratio (uACR) of 30–300 µg/mg, is an early marker of glomerular damage and a convenient, concentration-independent test used in routine practice, persistent elevation of which in diabetic and hypertensive patients signals a high risk of CKD progression and adverse cardiovascular events [9, 10].

Oxidative stress, a key pathophysiological driver of CKD progression, interacts with hemodynamic and metabolic factors to accelerate tubular injury, interstitial fibrosis, and vascular damage [11]. Excess reactive oxygen species and associated inflammatory mediators, including interleukin-6 and tumor necrosis factor-α, contribute to sustained renal injury and endothelial dysfunction. This pathophysiologic framework supports antioxidant therapy as a rational adjunctive strategy for slowing CKD progression [12].

N-acetylcysteine (NAC) is an acetylated precursor of L-cysteine and glutathione. NAC plays a crucial role in replenishing intracellular glutathione and scavenging reactive oxygen species, leading to its antioxidant and anti-inflammatory effects [13, 14]. Systematic reviews and cohort studies in patients with CKD suggest that NAC improves estimated glomerular filtration rate (eGFR), and positively influences cardiovascular outcomes, while mitigating the long-term decline in kidney function [15–17]. Taurine (2-aminoethanesulfonic acid), a conditionally essential β-amino acid, possesses complementary nephroprotective properties, including antioxidant activity, regulation of cell volume and calcium homeostasis, and stabilization of cellular membranes in the kidneys [18, 19]. Experimental evidence indicates that a combination of NAC and taurine offers greater protection against oxidative and ischemic renal injury compared to either agent used independently [20–22].

Previous clinical studies in patients with diabetic nephropathy and early CKD reported reductions in microalbuminuria with NAC + taurine supplementation, particularly when initiated in earlier stages of the disease. A recent retrospective study in Indian patients with stages 1–3 CKD and diabetes and/or hypertension showed that 90 days of NAC + taurine was associated with an 11.77% reduction in mean uACR, with superior outcomes observed in earlier CKD stages [23]. Building on this evidence, this first real-world comparative study on the NAC + taurine combination was conducted exclusively in cardiology practices. The study aimed to evaluate the effectiveness and tolerability of NAC (150 mg) plus taurine (500 mg) as an adjunct to standard of care versus standard of care alone in adults with stages 1–3 CKD and type 2 diabetes mellitus (T2DM) and/or hypertension, using electronic medical record (EMR) data to generate practice-relevant evidence for clinical decision-making.

## Materials and Methods

### Study design and settings

This real-world, retrospective, multicentric, comparative study (CTRI/2025/08/093387; registered on 21 August, 2025) was conducted using anonymized and de-identified data of patients, retrieved from EMRs from cardiology practices of 14 sites across India between May 2024 and March 2025. EMR data of adult patients with stages 1–3 CKD and T2DM and/or hypertension were captured at baseline and after 12 ± 2 weeks of treatment initiation.

### Ethical considerations

This study followed the Declaration of Helsinki and Good Clinical Practice (GCP) guidelines, adhering to the standards set by the International Council for Harmonization (ICH), and the guidelines for Clinical Trials on Pharmaceutical Products in India as specified in the New Drugs and Clinical Trials Rules, 2019. Approval was obtained from an Independent Ethics Committee. Given the retrospective nature of the study involving aggregated, anonymized data from EMRs, informed consent was waived per the Indian Council of Medical Research (ICMR) 2017 guidelines.

### Participants

Male or female patients aged 18 years or older, with stage 1, 2, or 3 CKD defined by the National Kidney Foundation-Kidney Disease Outcomes Quality Initiative (NKF-KDOQI) criteria, diagnosed with T2DM and/or hypertension, prescribed standard of care with or without NAC (150 mg) and taurine (500 mg) combination by the treating cardiologist, and had uACR, serum creatinine, and eGFR recorded at baseline and at 12 ± 2 weeks were included. Patients were excluded if key study variables were missing or records were incomplete.

### Treatment

Data of patients who were prescribed a combination of NAC 150 mg and taurine 500 mg (Nefrosave^®^; Fourrts India Laboratories) plus standard of care (test) or standard of care alone (control) were retrieved. All participants underwent therapy (including recommendation for a low-protein diet when clinically indicated) as directed by a cardiologist for CKD and its associated comorbidities. Standard of care comprised routine guideline-directed management of CKD and associated diabetes and/or hypertension, including individualized pharmacological and non-pharmacological interventions prescribed according to the treating cardiologist’s clinical judgment and prevailing clinical practice guidelines (CPG). Owing to the real-world retrospective study design, treatment decisions were not protocol-mandated but reflected routine clinical practice across all participating centers.

### Endpoints

The primary endpoints of this study were changes in uACR and serum creatinine levels from baseline to 12 ± 2 weeks. Secondary endpoints comprised changes in eGFR over the same 12 ± 2-week period and the proportion of patients experiencing adverse events (AEs) and serious adverse events (SAEs). Information on family history, smoking, alcohol use, dietary habits, physical activity, and detailed concomitant medication use was not consistently available across participating centers and therefore could not be reliably included in the analysis. Although laboratory investigations were performed in accredited clinical laboratories using standardized reporting units, differences in analytical platforms across centers may have introduced some interlaboratory variability.

### Data collection

Data were collected for a duration of 12 ± 2 weeks following the initiation of treatment. Baseline measurements were defined as those recorded before the start of treatment. The following parameters were extracted: demographic information (age, sex, weight, height); vital signs (systolic and diastolic blood pressure (SBP and DBP), pulse rate); duration of CKD, T2DM, and hypertension; values for uACR, serum creatinine, and eGFR; glycated hemoglobin (HbA1c) and fasting blood sugar (FBS) levels for diabetic patients; any AEs.

### Sample size calculation

Sample size estimation was conducted using data published by Chiu et al (n = 7,668), which reported mean serum creatinine values of 1.82 mg/dL for NAC users compared to 2.12 mg/dL for non-users at 6 months, assuming a standard deviation (SD) of 1 resulting in an effect size of 0.3 mg/dL [15]. With an allocation ratio of 2:1 for test/reference, a desired statistical power of 80%, and a significance level of 5%, along with an anticipated attrition rate of 2%, the required sample size was determined to be approximately 450 patients, comprising 300 in the test arm and 150 in the reference arm.

### Hypothesis

The sample size calculation was based on a two-sided hypothesis test. The null hypothesis (H_0_) assumed that there would be no significant difference between the two treatment groups with respect to the primary outcome, whereas the alternative hypothesis (H_1_) assumed that a statistically significant difference would exist between the groups. It was a two-sided test.

### Statistical methods

All statistical analyses were conducted using IBM SPSS Statistics version 29.0.2.0 and R 4.5.2, in accordance with ICH E9 guidelines for statistical principles in clinical trials. Continuous variables were summarized as mean ± SD, along with median, minimum, maximum, and interquartile range (IQR; Q1, Q3). Categorical variables were presented as frequencies and percentages. Repeated-measures analysis of variance (ANOVA) was used to evaluate changes in the uACR across study visits. Repeated-measures analysis of covariance (ANCOVA) was subsequently performed to estimate adjusted mean differences after controlling for age, sex, height, weight, smoking and alcohol status, concomitant medication use (sodium–glucose cotransporter-2 (SGLT2) inhibitors, angiotensin-converting enzyme (ACE) inhibitors/angiotensin II receptor blockers (ARBs), mineralocorticoid receptor antagonists (MRAs), glucagon-like peptide-1 (GLP-1) receptor agonist), history of T2DM and hypertension, duration of CKD, baseline serum creatinine, and baseline eGFR. Univariate ANCOVA was used to compare the mean difference between the groups. The Mann–Whitney U test was utilized for between-group comparisons of continuous variables, due to the non-normal distribution of the data. The Wilcoxon signed-rank test was employed for within-group comparisons from baseline to follow-up. For categorical variables and proportions, the Chi-square test/Fisher’s exact test was applied. Between-group differences were reported as median differences with 95% confidence interval (CI). The study was designed to test the null hypothesis that there was no significant difference between the two study groups against the alternative hypothesis that a significant difference existed between the groups. All statistical tests were two-sided, and a P-value < 0.05 was considered statistically significant.

## Results

**Figure 1:**
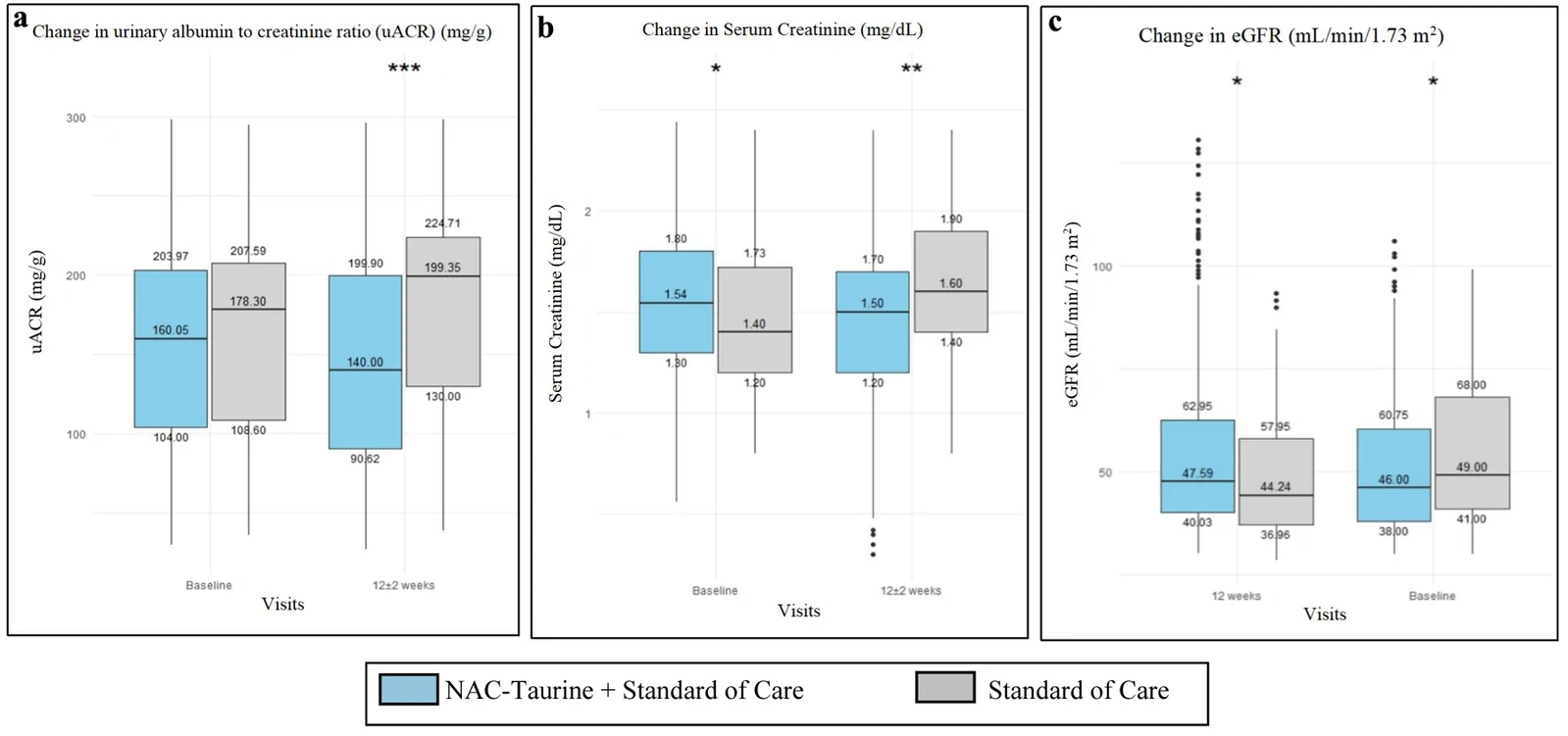
Median comparison between NAC + taurine plus standard of care versus standard of care alone from baseline to 12 ± 2 weeks for (a) change in uACR (mg/g), (b) change in serum creatinine (mg/dL), and (c) change in eGFR (mL/min/1.73 m^2^). NAC: N-acetylcysteine; uACR: albumin-to-creatinine ratio; eGFR: estimated glomerular filtration rate.

A total of 450 patients were included in the analysis, with 300 patients in the NAC + taurine plus standard-of-care group and 150 patients in the standard-of-care group. The detailed baseline demographic and clinical characteristics are summarized in Table 1. The mean age was 59.79 (± 12.30) years in the test group and 56.53 (± 13.80) years in the control. The mean body weight was comparable between the groups (test, 68.22 (± 9.63) kg; control, 68.77 (± 8.59) kg), as was the mean height (test, 165.74 (± 7.55) cm; control, 166.01 (± 7.33) cm). For clinical interpretation, normal renal function is characterized by eGFR ≥ 90 mL/min/1.73 m^2^ and uACR < 30 mg/g (Kidney Disease: Improving Global Outcomes (KDIGO) 2024 [24]). Following 12 ± 2 weeks of treatment, the NAC + taurine plus standard-of-care group demonstrated a significant reduction in mean baseline uACR (−8.61%; mean difference: −13.76 µg/mg; 95% CI: −15.27 to −12.26; P < 0.001), whereas the standard-of-care group showed a significant increase (+11.26%; mean difference: 18.68 µg/mg; 95% CI: 16.54 to 20.81; P < 0.001). After adjustment for baseline demographic and clinical characteristics, including concomitant nephroprotective therapies, the reduction in uACR remained significant in the NAC + taurine group (adjusted mean difference, δ = −13.76 µg/mg; 95% CI: −15.25 to −12.27; P < 0.001), while the standard-of-care group continued to demonstrate a significant increase (δ = 18.67 µg/mg; 95% CI: 16.50 to 20.84; P < 0.001). The adjusted between-group difference also remained highly significant in favor of NAC + taurine supplementation (δ = −33.01 µg/mg; 95% CI: −35.74 to −30.28; P < 0.001) (Table 2 and Fig. 1a). Accordingly, improved renal function is reflected by an increase in eGFR together with reductions in serum creatinine and uACR. At baseline, the use of guideline-directed nephroprotective therapies was documented in both treatment groups. These concomitant therapies were included as covariates in the repeated-measures ANCOVA to account for their potential influence on renal outcomes. None of the concomitant nephroprotective therapies showed a statistically significant association with renal outcomes in the adjusted analysis.

**Table 1:** Baseline Demographics and Clinical Characteristics

Parameters	NAC + taurine + standard of care	Standard of care	P-value
Overall patients, n	300	150	-
Age (years), mean ± SD	59.79 ± 12.30	56.53 ± 13.80	0.017*
Weight (kg), mean ± SD	68.22 ± 9.63	68.77 ± 8.59	0.374
Height (cm), mean ± SD	165.74 ± 7.55	166.01 ± 7.33	0.998
Sex			0.999
Male, n (%)	196 (65.33%)	98 (65.33%)	
Female, n (%)	104 (34.67%)	52 (34.67%)	
Stage of CKD			0.006**
Stage 1, n (%)	16 (5.33%)	19 (12.67%)	
Stage 2, n (%)	61 (20.33%)	33 (22.00%)	0.682
Stage 3, n (%)	223 (74.33%)	98 (65.33%)	0.047*
Systolic blood pressure (SBP) (mm Hg), mean ± SD	137.93 ± 16.53	141.39 ± 12.90	0.010*
Diastolic blood pressure (DBP) (mm Hg), mean ± SD	84.48 ± 7.97	85.85 ± 5.81	0.185
Pulse rate (beats per minute), mean ± SD	78.32 ± 6.50	77.01 ± 5.66	0.042*
SPO_2_ (%)			0.07
n	71	30	
Mean ± SD	96.31 ± 0.71	96.60 ± 0.89	
Temperature (°F)			0.25
n	11	9	
Mean ± SD	97.91 ± 0.48	97.70 ± 0.36	
Respiratory rate (breaths per minute)			0.301
n	11	11	
Mean ± SD	19.82 ± 2.27	20.64 ± 2.50	
Duration of CKD (years)			0.999
Number of patients with CKD (%)	300 (100%)	150 (100%)	
Mean ± SD	3.11 ± 2.76	1.93 ± 1.31	< 0.001***
Duration of hypertension (years)			0.027*
Number of patients with hypertension (%)	270 (90.00%)	144 (96.00%)	< 0.001***
Mean ± SD	9.56 ± 9.11	7.08 ± 9.46	
Duration of type 2 diabetes mellitus (T2DM) (years)			
Number of patients with T2DM (%)	188 (62.67%)	57 (38.00%)	< 0.001***
Mean ± SD	9.92 ± 9.10	11.67 ± 11.47	0.944
Both hypertension and type 2 diabetes mellitus, n (%)	158 (52.67%)	51 (34.00%)	< 0.001***
Alcohol status			
Non-drinker	259 (86.33%)	130 (86.67%)	0.922
Quit drinking	26 (8.67%)	10 (6.67%)	0.461
Casual drinking	5 (1.67%)	4 (2.67%)	0.619
Occasional drinker	7 (2.33%)	6 (4.00%)	0.320
Social drinker	3 (1.00%)	-	NA
Smoking status			
Non-smoker	244 (81.33%)	120 (80.00%)	0.735
Quit smoking	51 (17.00%)	20 (13.33%)	0.315
Casual smoking	1 (0.33%)	1 (0.33%)	0.499
Moderate smoking	1 (0.33%)	2 (1.33%)	0.248
Light smoking	3 (1.00%)	6 (4.00%)	0.243
Chain smoker	-	1 (0.67%)	NA

*P < 0.05, **P < 0.01, and ***P < 0.001. NAC: N-acetylcysteine; SD: standard deviation; CKD: chronic kidney disease; SPO_2_: oxygen saturation; NA: not available.

**Table 2:** Change in uACR (mg/g) Stratified by CKD Stages From Baseline to 12 ± 2 Weeks of Treatment in the Two Groups

	Baseline	12 ± 2 weeks	Mean difference from baseline to 12 ± 2 weeks, Δ (Δ%) (95% CI), P-value^###^	Adjusted mean difference from baseline to 12 ± 2 weeks, δ (95% CI), P-value^#^	Median of difference from baseline to 12 ± 2 weeks (95% CI), P-value^##^
Overall					
NAC + taurine + standard of care (n = 300)					
Mean ± SD, µg/mg	159.87 ± 65.37	146.11 ± 64.89	−13.76 (−8.61%), (−15.27, −12.26), < 0.001***	−13.76 (−15.25, −12.27), < 0.001***	−12.40 (−14.00, −10.80), < 0.001***
Median (min, max)	160.05 (30.00, 298.05)	140.00 (27.00, 295.60)			
Q1, Q3	104.00, 203.97	90.62, 199.90			
Standard of care (n = 150)			18.68 (11.26%) (16.54, 20.81), < 0.001***	18.67 (16.50, 20.84), < 0.001***	18.10 (16.63, 19.66), < 0.001***
Mean ± SD, µg/mg	165.85 ± 58.71	184.53 ± 58.64			
Median (min, max)	178.30 (36.00, 294.56)	199.35 (39.00, 298.10)			
Q1, Q3	108.60, 207.59	130.00, 224.71			
Between-group difference			−32.44 (−35.05, −29.83), < 0.001***	−33.01 (−35.74, −30.28), < 0.001***	−30.34 (−32.60, −28.00), < 0.001***
Stage 1 CKD					
NAC + taurine + standard of care (n = 16)					
Mean ± SD, µg/mg	146.16 ± 54.77	117.75 ± 59.70	−28.41 (−19.44%) (−33.71, −23.11), < 0.001***	−24.11 (−33.74, −14.49), < 0.001***	−28.11 (−35.35, −21.89), < 0.001***
Median (min, max)	144.10 (85.00, 284.88)	110.15 (50.10, 280.00)			
Q1, Q3	98.50, 179.65	66.28, 150.52			
Standard of care (n = 19)			24.85 (19.75%) (19.98, 29.71), < 0.001***	21.23 (12.90, 29.57), < 0.001***	24.31 (20.21, 29.39), < 0.001***
Mean ± SD, µg/mg	125.84 ± 31.58	150.69 ± 33.86			
Median (min, max)	121.67 (88.50, 198.54)	140.14 (109.80, 220.36)			
Q1, Q3	102.04, 148.30	122.98, 180.10			
Between-group difference			−53.26 (−60.45, −46.06), < 0.001***	−45.65 (−62.46, −28.84), < 0.001***	−52.55 (−61.00, −45.20), < 0.001***
Stage 2 CKD					
NAC + taurine + standard of care (n = 61)					
Mean ± SD, µg/mg	155.61 ± 49.98	131.18 ± 46.86	−24.43 (−15.70%) (−27.99, −20.87), < 0.001***	−25.05 (−28.72, −21.38), < 0.001***	−23.11 (−27.00, −19.69), < 0.001***
Median (min, max)	156.87 (44.00, 280.10)	124.00 (30.00, 250.16)			
Q1, Q3	114.65, 185.56	94.47, 154.35			
Standard of care (n = 33)			19.91 (10.77%) (15.07, 24.75), < 0.001***	21.05 (15.76, 26.35), < 0.001***	19.41 (15.22, 23.29), < 0.001***
Mean ± SD, µg/mg	184.89 ± 59.13	204.80 ± 57.25			
Median (min, max)	191.28 (94.60, 288.30)	210.40 (120.00, 298.00)			
Q1, Q3	120.00, 222.36	130.50, 245.55			
Between-group difference			−44.34 (−50.36, −38.34), < 0.001***	−48.46 (−55.70, −41.22), < 0.001***	−42.28 (−48.46, −37.00), < 0.001***
Stage 3 CKD					
NAC + taurine + standard of care (n = 223)					
Mean ± SD, µg/mg	162.02 ± 69.68	152.22 ± 68.42	−9.80 (−6.05%) (−11.32, −8.27), < 0.001***	−9.42 (−10.93, −7.90), < 0.001***	−8.78 (−10.17, −7.52), < 0.001***
Median (min, max)	166.10 (30.00, 298.05)	150.00 (27.00, 295.60)			
Q1, Q3	104.00, 208.90	96.00, 202.20			
Standard of care (n = 98)			17.06 (10.20%) (14.77, 19.36), < 0.001***	16.21 (13.87, 18.55), < 0.001***	16.52 (15.00, 18.26), < 0.001***
Mean ± SD, µg/mg	167.20 ± 59.31	184.26 ± 60.20			
Median (min, max)	186.78 (36.00, 294.56)	200.10 (39.00, 298.10)			
Q1, Q3	107.00, 208.09	121.15, 226.62			
Between-group difference			−26.86 (−29.62, −24.10), < 0.001***	−26.08 (−28.97, −23.18), < 0.001***	−25.22 (−27.58, −23.04), < 0.001***

Statistical tests used: ^#^Mann−Whitney U test; ^##^Wilcoxon signed-rank test; ^###^repeated-measures ANOVA; ***repeated-measures ANCOVA; between-group difference: univariate ANCOVA; level of significance, P < 0.001. uACR: urinary albumin-to-creatinine ratio; CI: confidence interval; NAC: N-acetylcysteine; SD: standard deviation; CKD: chronic kidney disease; Q1, Q3: first and third quartiles; ANOVA: repeated-measures analysis of variance; ANCOVA: repeated-measures analysis of covariance; min, max: minimum, maximum.

Baseline median serum creatinine levels were marginally higher in the test group compared to the control (1.54 vs. 1.47 mg/dL). Post-treatment for 12 ± 2 weeks, the test group exhibited significantly lower serum creatinine levels (1.46 mg/dL) compared to the control group (1.60 mg/dL). In fact, the test group demonstrated a significant reduction in serum creatinine, whereas the control group showed a significant increase (P < 0.001 for both). The between-group difference of −0.20 mg/dL was statistically significant (P < 0.001) and remained significant after adjustment for baseline covariates (Table 3 and Fig. 1b).

**Table 3:** Change in Serum Creatinine (mg/dL) Stratified by CKD Stages From Baseline to 12 ± 2 Weeks of Treatment in the Two Groups

	Baseline	12 ± 2 weeks	Median of difference from baseline to 12 ± 2 weeks (95% CI), P-value^##^	Median of difference between groups (95% CI), P-value^#^
Overall				
NAC + taurine + standard of care (n = 300)			−0.05 (−0.06, −0.05), < 0.001***	−0.20 (−0.20, −0.20), < 0.001***
Mean ± SD, mg/dL	1.54 ± 0.38	1.46 ± 0.43		
Median (min, max)	1.54 (0.56, 2.44)	1.50 (0.30, 2.40)		
Q1, Q3	1.30, 1.80	1.20, 1.70		
Standard of care (n = 150)			0.15 (0.11, 0.15), < 0.001***	
Mean ± SD, mg/dL	1.47 ± 0.37	1.60 ± 0.35		
Median (min, max)	1.40 (0.80, 2.40)	1.60 (0.80, 2.40)		
Q1, Q3	1.20, 1.73	1.40, 1.90		
Stage 1 CKD				
NAC + taurine + standard of care (n = 16)			−0.20 (−0.29, −0.09), 0.001**	−0.36 (−0.48, −0.26), < 0.001***
Mean ± SD, mg/dL	0.85 ± 0.12	0.66 ± 0.19		
Median (min, max)	0.90 (0.56, 1.00)	0.69 (0.40, 1.00)		
Q1, Q3	0.80, 0.91	0.50, 0.82		
Standard of care (n = 19)			2.00 (0.15, 2.50), < 0.001***	
Mean ± SD, mg/dL	1.01 ± 0.11	1.19 ± 0.16		
Median (min, max)	1.10 (0.80, 1.10)	1.20 (0.80, 1.40)		
Q1, Q3	0.90, 1.10	1.10, 1.30		
Stage 2 CKD				
NAC + taurine + standard of care (n = 61)			−0.13 (−0.18, −0.10), < 0.001***	−0.30 (−0.35, −0.21), < 0.001***
Mean ± SD, mg/dL	1.16 ± 0.21	1.00 ± 0.28		
Median (min, max)	1.20 (0.80, 1.60)	1.00 (0.30, 1.50)		
Q1, Q3	0.98, 1.31	0.80, 1.20		
Standard of care (n = 33)			0.15 (0.12, 0.20), < 0.001***	
Mean ± SD, mg/dL	1.16 ± 0.16	1.33 ± 0.20		
Median (min, max)	1.20 (0.80, 1.40)	1.40 (1.00, 1.70)		
Q1, Q3	1.10, 1.30	1.10, 1.50		
Stage 3 CKD				
NAC + taurine + standard of care (n = 223)			−0.05 (−0.05, −0.03), < 0.001***	−0.17 (−0.20, −0.13), < 0.001***
Mean ± SD, mg/dL	1.69 ± 0.29	1.65 ± 0.30		
Median (min, max)	1.70 (1.08, 2.44)	1.60 (1.00, 2.40)		
Q1, Q3	1.50, 1.89	1.40, 1.80		
Standard of care (n = 98)			0.11 (0.10, 0.14), < 0.001***	
Mean ± SD, mg/dL	1.66 ± 0.30	1.78 ± 0.28		
Median (min, max)	1.60 (1.10, 2.40)	1.80 (1.10, 2.40)		
Q1, Q3	1.40, 1.89	1.58, 2.00		

Statistical tests used: ^#^Mann–Whitney U test; ^##^Wilcoxon signed-rank test; level of significance, ***P < 0.001. CI: confidence interval; NAC: N-acetylcysteine; SD: standard deviation; CKD: chronic kidney disease; Q1, Q3: first and third quartiles; min, max: minimum, maximum.

The test group demonstrated significant improvement in eGFR by 2.06 mL/min/1.73 m^2^ (P < 0.001), whereas the control group showed a significant decline by 5.47 mL/min/1.73 m^2^ (P < 0.001). The between-group difference was statistically significant (P < 0.001) and remained significant after adjustment for baseline covariates (Table 4 and Fig. 1c).

**Table 4:** Change in eGFR (mL/min/1.73 m^2^) Stratified by CKD Stages From Baseline to 12 ± 2 Weeks of Treatment in the Two Groups

	Baseline	12 ± 2 weeks	Median of difference from baseline to 12 ± 2 weeks (95% CI), P-value^##^	Median of difference between groups (95% CI), P-value^#^
Overall				
NAC + taurine + standard of care (n = 300)			2.06 (1.60, 2.71), < 0.001***	7.51 (6.52, 8.92), < 0.001***
Mean ± SD, mL/min/1.73 m^2^	50.62 ± 17.19	54.97 ± 22.42		
Median (min, max)	46.00 (30.00, 106.00)	47.59 (30.13, 130.60)		
Q1, Q3	38.00, 60.75	40.03, 62.95		
Standard of care (n = 150)			−5.47 (−6.75, −4.54), < 0.001***	
Mean ± SD, mL/min/1.73 m^2^	55.30 ± 19.18	48.62 ± 15.24		
Median (min, max)	49.00 (30.00, 99.00)	44.24 (28.39, 93.19)		
Q1, Q3	41.00, 68.00	36.96, 57.95		
Stage 1 CKD				
NAC + taurine + standard of care (n = 16)			10.05 (3.97, 17.20), < 0.001***	25.97 (19.89, 33.65), < 0.001***
Mean ± SD, mL/min/1.73 m^2^	97.00 ± 4.62	108.07 ± 11.81		
Median (min, max)	96.00 (91.00, 106.00)	107.12 (93.68, 130.60)		
Q1, Q3	92.50, 101.25	96.13, 117.04		
Standard of care (n = 19)				
Mean ± SD, mL/min/1.73 m^2^	93.00 ± 2.38	76.92 ± 8.02	−17.40 (−20.56, −11.73), < 0.001***	
Median (min, max)	93.00 (90.00, 99.00)	74.39 (66.53, 93.19)		
Q1, Q3	91.00, 95.00	70.63, 84.05		
Stage 2 CKD				
NAC + taurine + standard of care (n = 61)			9.96 (7.33, 13.44), < 0.001***	19.67 (16.00, 24.20), < 0.001***
Mean ± SD, mL/min/1.73 m^2^	69.34 ± 7.87	81.56 ± 16.79		
Median (min, max)	66.00 (60.00, 86.00)	77.43 (58.37, 128.35)		
Q1, Q3	62.00, 76.50	67.37, 92.98		
Standard of care (n = 33)			−10.22 (−12.70, −7.76), < 0.001***	
Mean ± SD, mL/min/1.73 m^2^	68.94 ± 6.34	58.71 ± 8.39		
Median (min, max)	69.00 (61.00, 86.00)	58.24 (44.38, 83.84)		
Q1, Q3	64.00, 71.50	53.18, 62.32		
Stage 3 CKD				
NAC + taurine + standard of care (n = 223)			1.26 (0.98, 1.51), < 0.001***	11.39 (9.58, 12.79), < 0.001***
Mean ± SD, mL/min/1.73 m^2^	42.17 ± 7.54	43.89 ± 8.40		
Median (min, max)	42.00 (30.00, 59.00)	42.83 (30.13, 70.87)		
Q1, Q3	36.00, 48.00	37.15, 49.77		
Standard of care (n = 98)			−3.47 (−4.07, −2.95), < 0.001***	
Mean ± SD, mL/min/1.73 m^2^	43.40 ± 8.14	39.73 ± 7.08		
Median (min, max)	43.00 (30.00, 59.00)	38.67 (28.39, 57.88)		
Q1, Q3	37.00, 49.00	34.90, 44.08		

Statistical tests used: ^#^Mann–Whitney U test; ^##^Wilcoxon signed-rank test; level of significance, ***P < 0.001. eGFR: estimated glomerular filtration rate; CI: confidence interval; NAC: N-acetylcysteine; SD: standard deviation; CKD: chronic kidney disease; Q1, Q3: first and third quartiles; min, max: minimum, maximum.

**Figure 2:**
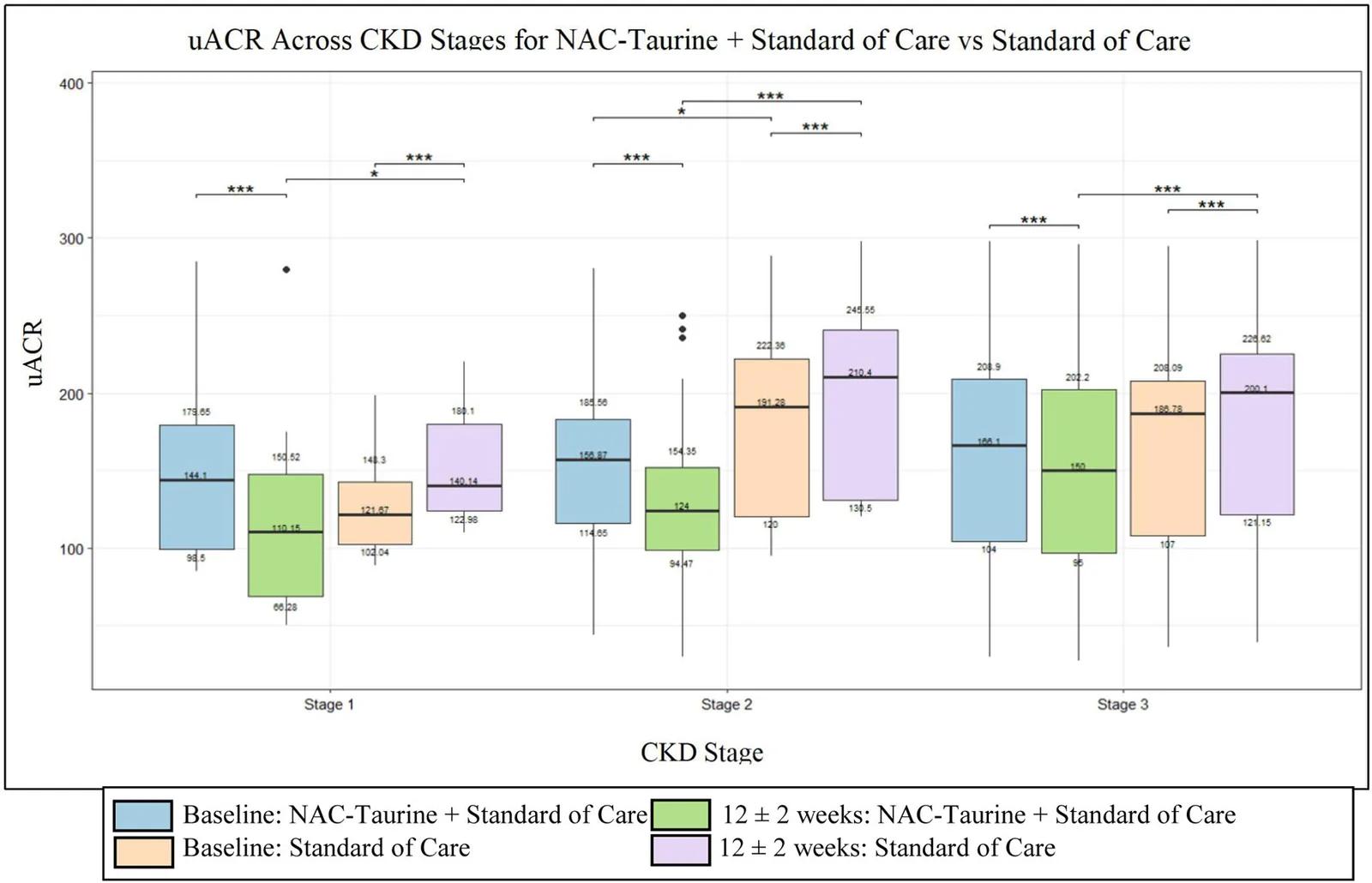
Median comparison between NAC + taurine plus standard of care versus standard of care alone from baseline to 12 ± 2 weeks stratified by CKD stages for change in uACR (mg/g). NAC: N-acetylcysteine; uACR: albumin-to-creatinine ratio; CKD: chronic kidney disease. Level of significance, *P < 0.05, ***P < 0.001.

**Figure 3:**
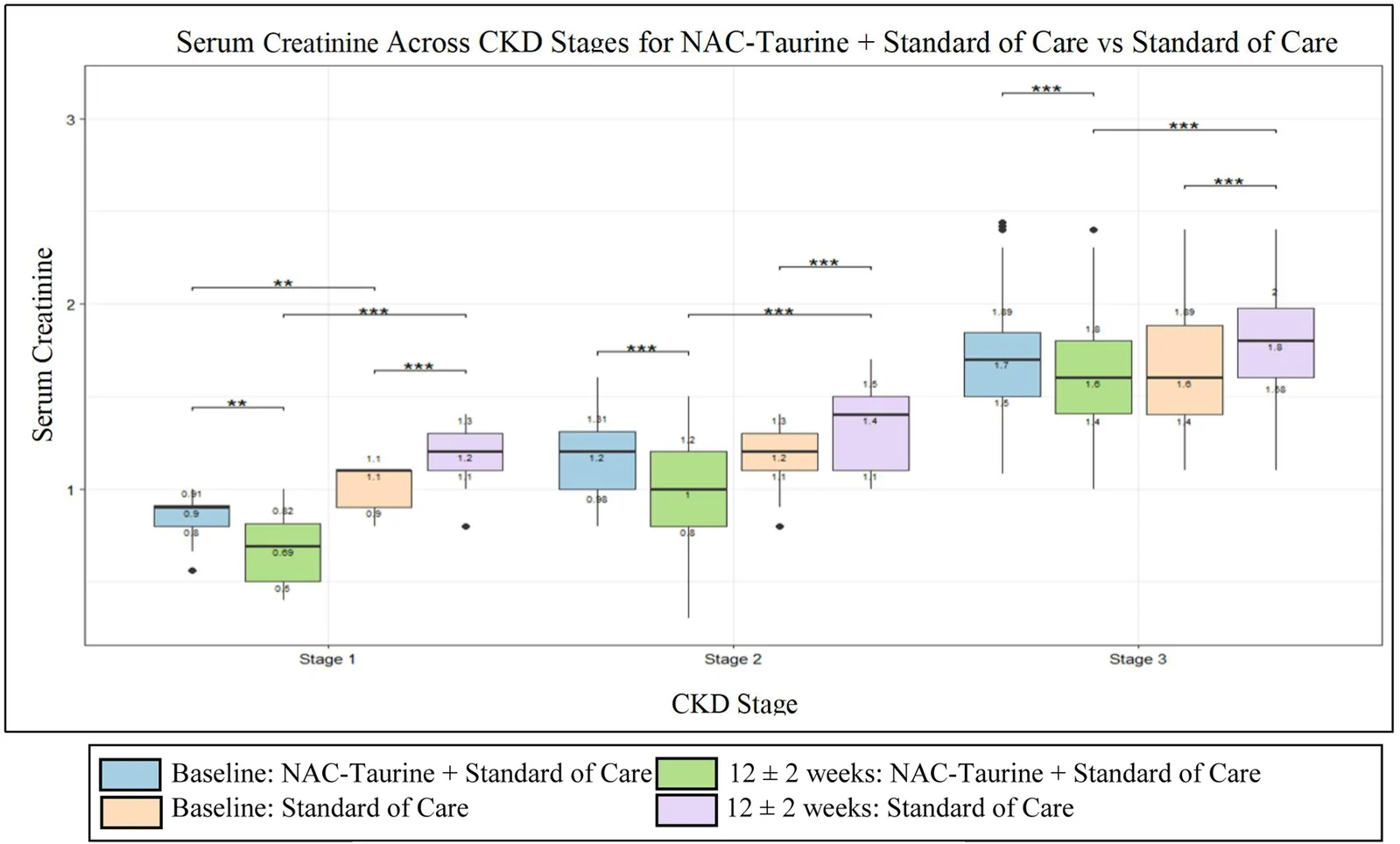
Median comparison between NAC + taurine plus standard of care versus standard of care alone from baseline to 12 ± 2 weeks stratified by CKD stages for change in serum creatinine (mg/dL). NAC: N-acetylcysteine; CKD: chronic kidney disease. Level of significance, *P < 0.05, ***P < 0.001.

**Figure 4:**
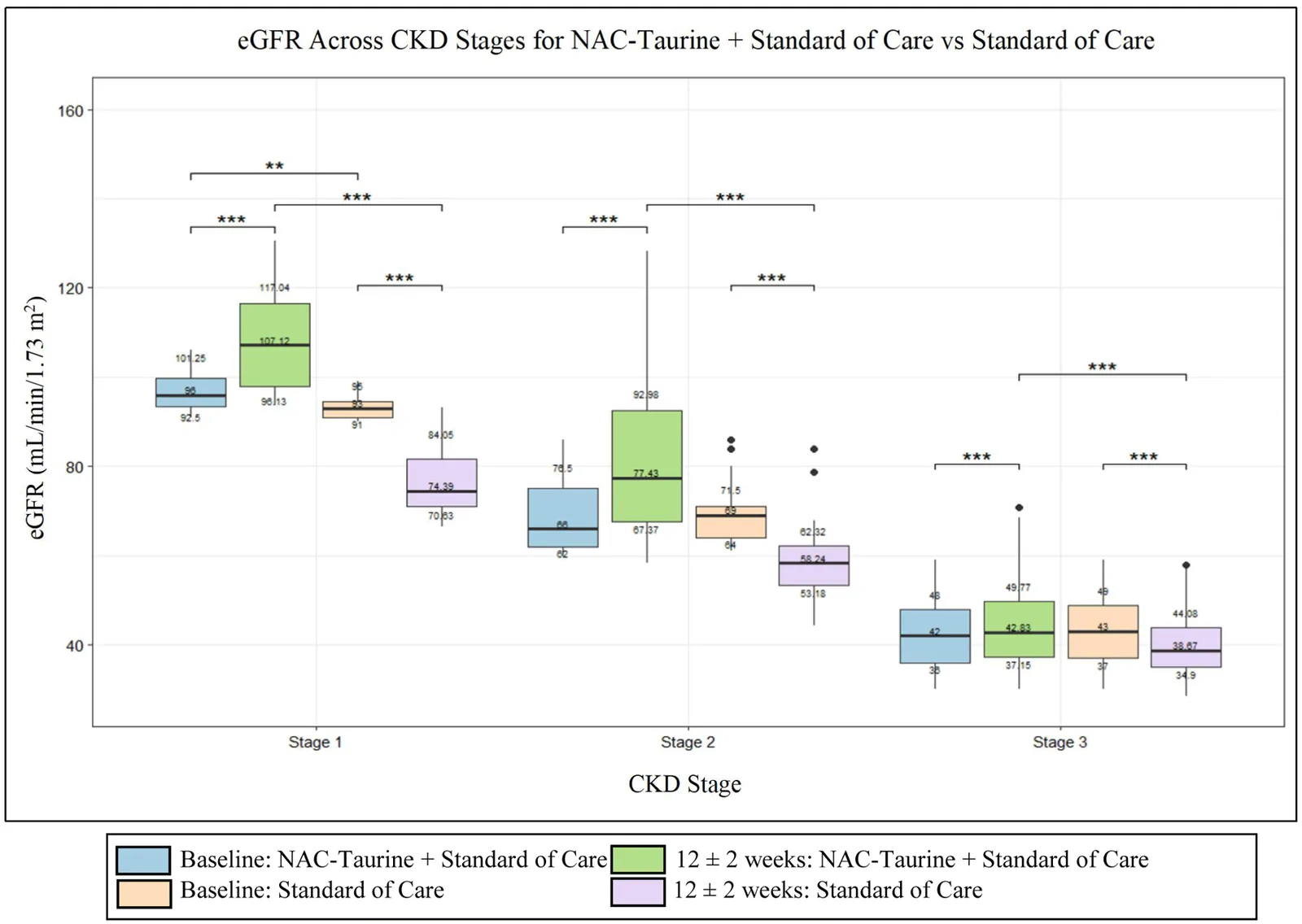
Median comparison between NAC + taurine plus standard of care versus standard of care alone from baseline to 12 ± 2 weeks stratified by CKD stages for change in eGFR (mL/min/1.73 m^2^). NAC: N-acetylcysteine; CKD: chronic kidney disease; eGFR: estimated glomerular filtration rate. Level of significance, *P < 0.05, ***P < 0.001.

Across CKD stages 1–3, adjunctive NAC + taurine plus standard of care consistently improved uACR, serum creatinine, and eGFR compared with standard of care alone, indicating a robust nephroprotective effect across the disease spectrum. The greatest benefit was observed in early CKD stages, where NAC + taurine not only reduced albuminuria but also decreased serum creatinine levels and improved eGFR, while patients on standard of care alone showed progressive worsening of these markers. Even in stage 3 CKD, NAC + taurine plus standard of care attenuated further deterioration of uACR, serum creatinine, and eGFR relative to standard of care alone, supporting its role in delaying renal decline in more advanced disease (Tables 2–4 and Figs. 2–4).

**Figure 5:**
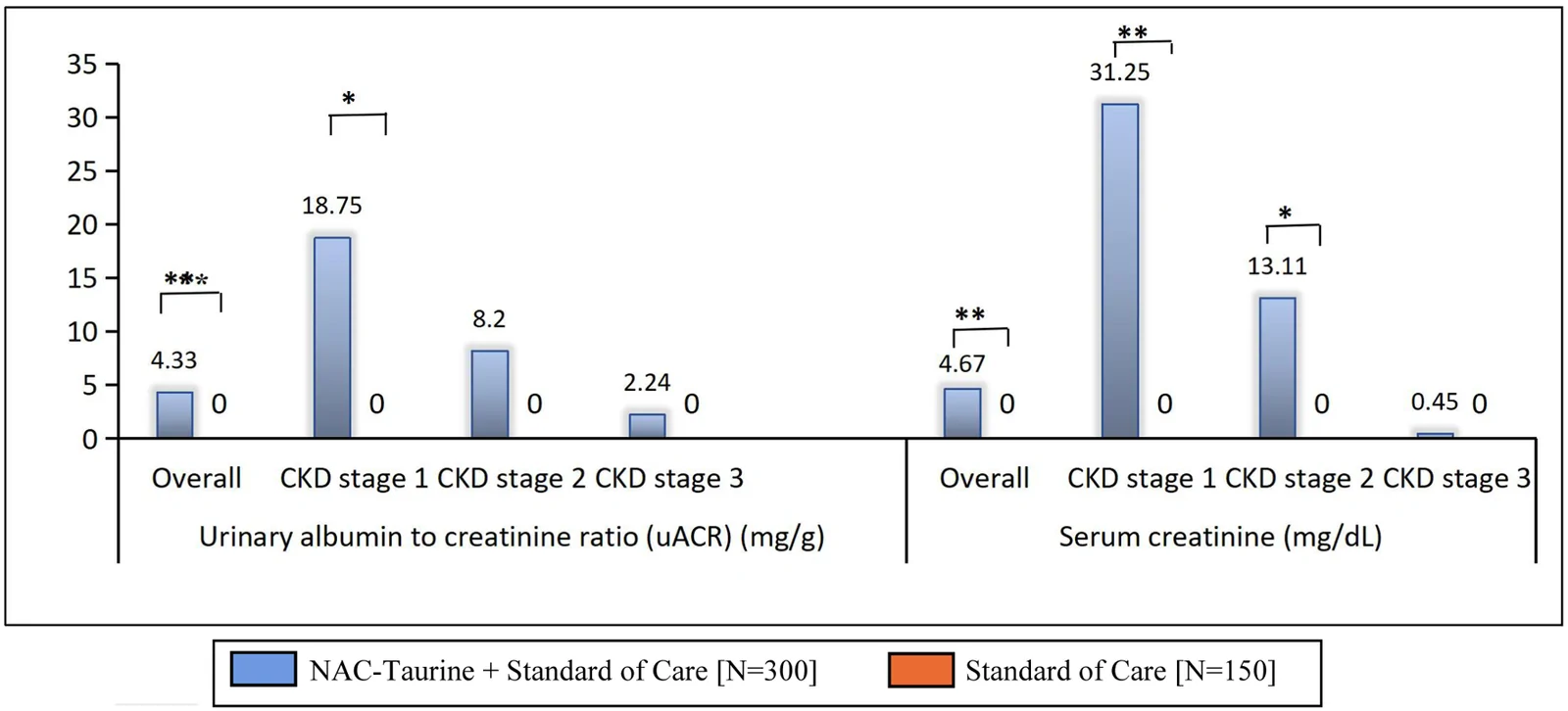
Proportions of patients with ≥ 30% improvement in uACR and serum creatinine from baseline to 12 ± 2 weeks. uACR: urinary albumin-to-creatinine ratio. Level of significance, *P < 0.05, **P < 0.01.

A significantly higher proportion of patients in the treatment group achieved ≥ 30% improvement in uACR compared with controls (P = 0.009). Similarly, ≥ 30% improvement in serum creatinine was observed exclusively in the test group (P = 0.007). Subgroup analysis demonstrated consistent benefits across all CKD stages, with greater absolute reductions observed in earlier stages. Stage 1 CKD patients in the test group showed the highest median reduction in uACR and serum creatinine levels, followed by stages 2 and 3 (Fig. 5).

AEs were reported in nine out of 300 patients (3.00%) in the NAC + taurine plus standard-of-care group compared with none in the control group. All AEs were classified as related to treatment, of mild severity, and self-limiting. The events comprised nausea (n = 5), vomiting (n = 2), diarrhea (n = 1), and abdominal pain (n = 1). No SAEs or treatment discontinuations were reported.

Additionally, in patients with T2DM (n = 245), HbA1c levels decreased significantly in both groups, with no significant difference between them (P = 0.517). FBS decreased in both groups. For hypertensive patients (n = 414), both groups showed significant reductions in SBP and DBP; however, there were no significant between-group differences (SBP: P = 0.810; DBP: P = 0.439).

## Discussion

This first, retrospective, multicentric, real-world, comparative study conducted exclusively in cardiology practices demonstrated that compared to standard of care alone, adjunctive NAC + taurine combination plus standard-of-care therapy significantly improved renal function in adults with stages 1–3 CKD having concurrent diabetes and/or hypertension. The combination therapy resulted in significant reductions in microalbuminuria and serum creatinine levels, along with increases in eGFR, despite the test group having a high baseline comorbidity burden. In contrast, standard of care alone resulted in worsening of the renal parameters, declining patient outcomes. Viswanathan et al demonstrated approximately 50% reduction in uACR with NAC + taurine combination therapy in patients with diabetes and microalbuminuria [25]. In our study, the observed between-group difference in mean uACR of −32.44 µg/mg is clinically significant, as uACR is a crucial marker of glomerular injury and a well-established predictor of kidney and cardiovascular outcomes [26–28]. Importantly, this treatment effect remained robust after adjustment for baseline covariates, with an adjusted between-group mean difference (δ) of −33.01 µg/mg (95% CI: −35.74 to −30.28; P < 0.001), indicating that the reduction in albuminuria was independent of baseline clinical differences between the treatment groups. Even modest reductions in uACR have been linked to decreased risk of CKD progression and major adverse cardiovascular events (MACEs) in previous studies [28, 29]. Although the observed absolute reduction was modest, statistical significance does not necessarily imply clinical significance. Therefore, these findings should be interpreted cautiously until confirmed in prospective studies with long-term clinical endpoints. The Losartan Intervention for Endpoint reduction in hypertension (LIFE) [30, 31] and Heart Outcomes Prevention Evaluation (HOPE) [32] studies demonstrated continuous relationships between uACR and cardiovascular morbidity across the entire spectrum of albuminuria, underscoring the importance of interventions targeting this marker.

Cardiologists must actively monitor uACR and serum creatinine because both are powerful, independent predictors of adverse cardiovascular as well as renal outcomes in patients with diabetes and hypertension. Even modest, sustained elevations in these markers are associated with higher risks of heart failure, MACEs, and accelerated CKD progression, underscoring their value as early warning signals rather than late-stage indicators. Consequently, relying on standard of care alone may be insufficient when these markers remain abnormal. Timely initiation of additional nephroprotective strategies such as NAC-taurine, alongside guideline-directed cardio-renal therapy, is warranted to modify long-term cardio-renal risk rather than simply treating established complications.

The concurrent decline in serum creatinine and improvement in eGFR support a true nephroprotective effect rather than isolated modification of albuminuria. These findings corroborate the 3-year retrospective cohort study by Chiu et al [15] and other studies [23, 33, 34], which showed slower CKD progression and better preservation of eGFR with NAC in routine practice. A meta-analysis by Ye et al demonstrated that NAC consistently lowered oxidative stress markers in CKD patients. However, clinical improvements in kidney function and patient outcomes remain inconclusive [17]. The current comparative design extends that evidence by demonstrating that NAC + taurine not only stabilizes kidney function, but also counters the spontaneous worsening of albuminuria and creatinine observed under standard of care alone.

The biological plausibility of these findings is substantial. Beyond renal protection, CKD is increasingly recognized as a component of the broader cardio–renal–metabolic (CRM) syndrome, characterized by persistent oxidative stress, systemic inflammation, endothelial dysfunction, and subclinical myocardial impairment. Emerging evidence suggests that advanced echocardiographic techniques, particularly speckle-tracking echocardiography, can detect early myocardial dysfunction before conventional measures of cardiac function become abnormal, thereby providing incremental cardio-renal risk stratification. Although cardiac imaging parameters were beyond the scope of the present retrospective study, future prospective investigations evaluating NAC + taurine therapy may incorporate these sensitive imaging modalities to determine whether improvements in oxidative stress and endothelial function are accompanied by measurable cardiovascular benefits. NAC replenishes intracellular glutathione, reduces oxidative stress, and diminishes inflammatory signaling within renal tissue [35]. Meanwhile, taurine provides complementary effects that are antioxidative, osmoregulatory, and membrane-stabilizing [18]. Experimental research suggests a synergistic protective effect when these two agents are co-administered, with combination therapy proving more effective at limiting oxidative damage and tubular injury than either agent alone [20].

The CKD stage-wise analyses presented in this study demonstrated significant between-group differences in uACR in stage 1 compared to stage 3 CKD, aligning with the recommendations of the KDIGO 2024 guidelines on early intervention in CKD [36, 37]. This indicates that antioxidant-based nephroprotection may be most effective when initiated before the onset of substantial structural damage [38]. In this study, uACR, serum creatinine, and eGFR data demonstrate that standard-of-care therapy alone failed to prevent short-term deterioration of renal function, while adjunctive NAC + taurine combination plus standard of care attenuated CKD progression and improved renal outcomes across the CKD spectrum. The systematic review by Colombijn et al evaluated antioxidants in CKD and found moderate certainty evidence that antioxidants reduce progression to kidney failure and may improve kidney function. However, the review noted substantial heterogeneity among interventions and limited evidence for individual agents [39]. The current study addresses this gap by providing focused real-world evidence for a specific combination therapy in a well-defined patient population.

The favorable safety profile observed in this study aligns with the established safety data for both NAC and taurine [17, 23]. All events were self-limiting without necessitating treatment discontinuation, supporting the suitability of this combination for long-term adjunctive therapy.

The comparable reductions in blood pressure and glycemic parameters between groups suggest that the observed renal benefits are independent of hemodynamic or metabolic effects. Both treatment arms demonstrated significant improvements in SBP and HbA1c, likely reflecting optimization of the standard of care during the follow-up period. The dissociation between renal outcomes and cardiovascular risk factors implies direct nephroprotective mechanisms mediated by antioxidant activity. The magnitude and direction of the effect observed here align with prior clinical reports of NAC + taurine combination in patients with diabetes and CKD characterized by microalbuminuria, while the present multicenter, EMR-based comparative dataset offers stronger external validity and a clearer counterfactual explanation than earlier single-arm series. In the broader context of antioxidant therapy in CKD, which has shown heterogeneous results and only moderate-certainty benefits in meta-analyses, this study provides focused, real-world support for a specific, fixed-dose combination in a well-defined, high-risk population. The low incidence of mild, self-limiting gastro-intestinal AEs and absence of serious toxicity reinforce the favorable benefit–risk profile of NAC + taurine as an adjunct to contemporary cardio-renal standard of care.

### Limitations

This study has several limitations inherent to its retrospective observational design. The absence of randomization introduces the possibility of selection bias and confounding by indication, particularly because patients receiving adjunctive NAC + taurine therapy had a higher prevalence of T2DM and more advanced CKD at baseline. Although multivariable analyses were performed to adjust for measured baseline differences, residual confounding from unmeasured or incompletely recorded variables cannot be excluded. Furthermore, if patients with incomplete baseline or follow-up data were excluded from the analysis, this may have introduced additional selection bias. The 12-week follow-up period was sufficient to assess short-term changes in the evaluated kidney function and albuminuria parameters but did not permit assessment of the durability of treatment effects or long-term kidney and cardiovascular outcomes. Hospitalization, progression to kidney failure, initiation of dialysis or other kidney replacement therapy, major adverse cardiovascular events, and mortality were not consistently documented across the participating centers and therefore could not be reliably evaluated. The study relied on routinely collected EMRs, which limited the availability and completeness of several clinically relevant variables. Although smoking status, alcohol consumption, medication history, and major comorbidities were available and included in the analysis, information on family history, dietary habits, physical activity, treatment adherence, medication dose changes, and certain concomitant therapies was not consistently captured. In particular, incomplete documentation of contemporary kidney and cardiovascular-protective therapies, such as SGLT2 inhibitors, finerenone, and GLP-1 agonists, may have resulted in residual treatment-related confounding. Laboratory investigations were performed across multiple centers using routine institutional laboratory platforms. Consequently, some interlaboratory variation in assay methods, calibration, and measurements cannot be excluded. Cystatin C measurements were unavailable, preventing the assessment of kidney function using a filtration marker independent of serum creatinine and limiting a more comprehensive evaluation of eGFR. Finally, the study population consisted exclusively of patients managed in cardiology practices across India. This setting may limit the generalizability of the findings to patients treated primarily in nephrology practices, other healthcare systems, different geographic regions, and populations with different ethnic, socio-economic, or clinical characteristics. Prospective, adequately powered, multicenter studies with longer follow-up, standardized laboratory assessments, systematic collection of concomitant therapies and adherence data, and predefined kidney and cardiovascular outcomes are needed to confirm the effectiveness, durability, and generalizability of adjunctive NAC + taurine therapy.

### Conclusions

The NAC + taurine combination added to standard of care improved renal outcomes, whereas standard of care alone was associated with worsening of key renal parameters, including uACR, serum creatinine, and eGFR. Across CKD stages 1–3, patients receiving NAC + taurine plus standard of care showed stabilization or improvement in albuminuria and creatinine, while those on standard of care alone exhibited progressive deterioration, underscoring the failure of standard therapy alone to contain renal decline. These findings support NAC + taurine combination as a promising adjunctive therapy to conventional cardio-renal therapies in diabetic and hypertensive patients with CKD.

Thus, NAC (150 mg) and taurine (500 mg) plus standard of care significantly reduced uACR and serum creatinine while improving eGFR compared with standard of care alone in diabetic and hypertensive patients with stages 1–3 CKD, with a favorable safety profile. These real-world findings support the potential role of the NAC + taurine combination as an effective nephroprotective adjunctive therapy. To further validate these encouraging real-world findings and establish their long-term clinical applicability, future prospective randomized controlled trials with extended follow-up periods and concrete cardio-renal endpoints are needed to corroborate the present findings. Research evaluating the combination therapy across diverse geographic populations and healthcare settings would enhance the evidence base. Furthermore, mechanistic studies elucidating the specific molecular pathways by which the NAC + taurine combination provides nephroprotection would aid in optimizing dosing regimens and identifying patient subgroups who are more likely to respond.

## Data Availability

Any inquiries regarding supporting data availability of this study should be directed to the corresponding author.

## Abbreviations

AEs: adverse events
ACE inhibitors: angiotensin-converting enzyme inhibitors
ARBs: angiotensin II receptor blockers
CI: confidence interval
CKD: chronic kidney disease
CRM: cardio–renal–metabolic
CTRI: Clinical Trials Registry-India
DBP: diastolic blood pressure
eGFR: estimated glomerular filtration rate
EMR: electronic medical record
FBS: fasting blood sugar
GCP: Good Clinical Practice
GLP-1 receptor agonists: glucagon-like peptide-1 receptor agonists
HbA1c: glycated hemoglobin
HOPE: Heart Outcomes Prevention Evaluation
ICH: International Council for Harmonization
ICMR: Indian Council of Medical Research
IQR: interquartile range
KDIGO: Kidney Disease, Improving Global Outcomes
LIFE: Losartan Intervention For Endpoint Reduction in Hypertension
MACEs: major adverse cardiovascular events
MRAs: mineralocorticoid receptor antagonists
NAC: N-acetylcysteine
NKF-KDOQI: National Kidney Foundation-Kidney Disease Outcomes Quality Initiative
Q1: Q3, first and third quartiles
SAEs: serious adverse events
SBP: systolic blood pressure
SD: standard deviation
SGLT2 inhibitors: sodium–glucose cotransporter-2 inhibitors
T2DM: type 2 diabetes mellitus
uACR: urinary albumin-to-creatinine ratio
